# Accounting for vaccines specificities in the Joint Clinical Assessment (JCA): a proposal for guiding principles

**DOI:** 10.1093/eurpub/ckac129.328

**Published:** 2022-10-25

**Authors:** E Beck, P D'Agostino, R Chapman, N Largeron, L Louette, U Sabale, K Schaible, S Quilici

**Affiliations:** 1 Value Evidence and Outcomes, GSK, Wavre, Belgium; 2 Global Market Access and Pricing, CSL Seqirus, München, Germany; 3 Evidence Synthesis, Modeling & Communication, Evidera, London, UK; 4 Global Market Access, Sanofi, Lyon, France; 5 Vaccines Europe, EFPIA, Brussels, Belgium; 6 CORE, MSD, Stockholm, Sweden

## Abstract

**Issue/problem:**

Vaccines are an important public health intervention protecting the population against infectious diseases. The value of vaccines is broad ranging from individual to societal as achieving community immunity protects the unvaccinated, minimizes the risk of outbreaks, reduces the emergence of antimicrobial resistance, and leads to broader societal benefits.

**Description of the problem:**

Vaccines’ market access processes are characterized by the development of recommendations by NITAGs followed by the assessment of health technology assessment (HTA) bodies in less than half of 27 EU member states. Despite that HTA for therapeutic drugs is well established, there is very limited experience in applying HTA methodologies to vaccines, especially for clinical assessments, as HTA methods and frameworks are traditionally geared toward therapeutics. However, following the adoption of the EU regulation on HTA, Joint Clinical Assessments (JCAs) of vaccines are expected.

**Results:**

To support a discussion on how to account for vaccine specificities in the JCA, Vaccines Europe has performed a project which aimed at developing a proposal for high-level guiding principles on processes and methodologies for clinical HTA for vaccines. The proposal is informed by findings of literature reviews on currently applied processes, methods, and clinical assessment frameworks of vaccines as well as the outcomes of an advisory board with scientific experts.

**Lessons:**

A proposal for high-level guiding principles for clinical HTA for vaccines is being developed based on both evidence and the advice from scientific experts which focuses on processes (e.g., horizon scanning, early advice, consideration of vaccine-specific expertise) and methods (e.g., unmet need, safety, efficacy/effectiveness, real-world evidence and technical characteristics of the technology). Lastly, the implementation of vaccines specificities in JCA represents a call for action.

